# Cross-border spread of *bla*_NDM-1_- and *bla*_OXA-48_-positive *Klebsiella pneumoniae*: a European collaborative analysis of whole genome sequencing and epidemiological data, 2014 to 2019

**DOI:** 10.2807/1560-7917.ES.2020.25.20.2000627

**Published:** 2020-05-21

**Authors:** Catherine Ludden, Felix Lötsch, Erik Alm, Narender Kumar, Karin Johansson, Barbara Albiger, Te-Din Huang, Olivier Denis, Anette M Hammerum, Henrik Hasman, Jari Jalava, Kati Räisänen, Laurent Dortet, Agnès B Jousset, Sören Gatermann, Sebastian Haller, Martin Cormican, Wendy Brennan, Maria Del Grosso, Monica Monaco, Leo Schouls, Ørjan Samuelsen, Mateja Pirš, Tjaša Cerar, Jésus Oteo-Iglesias, Maria Pérez-Vázquez, Karin Sjöström, Petra Edquist, Katie L Hopkins, Marc J Struelens, Daniel Palm, Dominique L Monnet, Anke Kohlenberg

**Affiliations:** 1 European Centre for Disease Prevention and Control, Stockholm, Sweden; 2 Department of Medicine, University of Cambridge, Cambridge, United Kingdom; 3 Belgian National Reference Center for Antibiotic-resistant Gram-negative bacilli, CHU UCL Namur, UCLouvain, Yvoir, Belgium; 4 Department of Bacteria, Parasites and Fungi, Statens Serum Institut, Copenhagen, Denmark; 5 Department of Health Security, Finnish Institute for Health and Welfare, Helsinki, Finland; 6 French National Reference Center for Antimicrobial Resistance, INSERM UMR 1184, Paris-Saclay University, Bicêtre hospital, Assistance Publique des Hôpitaux de Paris, Paris, France; 7 National Reference Centre for multidrug-resistant Gram-negative bacteria, Ruhr University Bochum, Bochum, Germany; 8 Robert Koch Institute, Department for Infectious Disease Epidemiology, Berlin, Germany; 9 National University of Ireland, Galway, Ireland; 10 Department of Infectious Diseases, Istituto Superiore di Sanità, Rome, Italy; 11 Infectious Diseases Research, Diagnostics and Laboratory Surveillance, National Institute for Public Health and the Environment, Bilthoven, the Netherlands; 12 Norwegian National Advisory Unit on Detection of Antimicrobial Resistance, University Hospital of North Norway, Tromsø, Norway; 13 Department of Pharmacy, Faculty of Health Sciences, UiT The Arctic University of Norway, Tromsø, Norway; 14 Institute of Microbiology and Immunology, Faculty of Medicine, University of Ljubljana, Ljubljana, Slovenia; 15 Laboratorio de Referencia e Investigación en Resistencia a Antibióticos e Infecciones Relacionadas con la Asistencia Sanitaria, Centro Nacional de Microbiología, Instituto de Salud Carlos III, Madrid, Spain; 16 Public Health Agency of Sweden, Stockholm, Sweden; 17 Healthcare Associated Infections and Antimicrobial Resistance Division, National Infection Service, Public Health England, London, United Kingdom

**Keywords:** carbapenem-resistant Enterobacterales, carbapenemase, OXA-48, NDM-1, *Klebsiella pneumonia*, surveillance, whole genome sequencing, cross-border import

## Abstract

Analysis of sequencing data for 143 *bla*_NDM-1_- and *bla*_OXA-48_-positive *Klebsiella pneumoniae* isolates from 13 European national collections and the public domain resulted in the identification of 15 previously undetected multi-country transmission clusters. For 10 clusters, cases had prior travel/hospitalisation history in countries outside of the European Union including Egypt, Iran, Morocco, Russia, Serbia, Tunisia and Turkey. These findings highlight the benefit of European whole genome sequencing-based surveillance and data sharing for control of antimicrobial resistance.

An alert regarding an outbreak of carbapenem-resistant *Klebsiella pneumoniae* carrying *bla*_NDM-1_ and *bla*_OXA-48_ carbapenemase-encoding genes was sent by Germany to European Union (EU)/European Economic Area (EEA) countries in October 2019 [1,2]. Since only limited whole genome sequencing (WGS) data on *bla*_NDM-1_- and *bla*_OXA-48_-positive *K. pneumoniae* were available in the public domain, national public health reference or equivalent expert laboratories from EU/EEA countries were invited to share WGS data from their national collections with the European Centre for Disease Prevention and Control (ECDC) to investigate the international dissemination of this epidemic strain. The analysis identified a Finnish case with an isolate closely related to the German outbreak strain and with an epidemiological link to St. Petersburg, Russia [1]. In addition, several other clusters of genetically related *bla*_NDM-1_- and *bla*_OXA-48_-positive *K. pneumoniae* unrelated to the German outbreak strain but affecting numerous EU/EEA countries were identified. The aim of this follow-up investigation was to characterise these clusters based on the integrated analysis of the WGS dataset on *bla*_NDM-1_*-* and *bla*_OXA-48_-positive *K. pneumoniae* submitted from 13 EU/EEA countries and additional epidemiological data.

## Definitions and origin of samples and data

A case was defined as an individual with an isolate of *K. pneumoniae* carrying both *bla*_NDM-1_ and *bla*_OXA-48_. An epidemiological link to a specific country was defined as either documented hospitalisation in or travel to this country within 6 months before isolation of the respective isolate. Samples and epidemiological information on *bla*_NDM-1_- and *bla*_OXA-48_-positive *K. pneumoniae* were provided by 13 EU/EEA countries by 5 December 2019: Belgium (n = 1), Denmark (n = 5), Finland (n = 2), France (n = 18), Germany (n = 39), Ireland (n = 1), Italy (n = 1), the Netherlands (n = 15), Norway (n = 1), Slovenia (n = 7), Spain (n = 3), Sweden (n = 8) and the UK (n = 16). Worldwide publicly available WGS data were retrieved from the National Centre for Biotechnology Information (NCBI) database on 23 November 2019. In total, WGS data from 143 isolates, i.e. 117 isolates from national collections and 26 publicly available isolates, were included in the analysis.

## Ethical statement

All data were anonymised and collected in accordance with the European Parliament and Council decisions on the epidemiological surveillance and control of communicable disease in the European Community. Ethical approval and informed consent were thus not required. 

## Whole genome sequencing analysis

Sequence data were processed as previously described [3]. The variants identified were used to create a pseudochromosome using a custom script (https://figshare.com/s/c7be54e5930e8b6a4103). The isolates were subjected to quality checks based on the highest proportion of reads mapping to *K. pneumoniae* obtained in Kraken, on sequencing coverage depth (range: 18–280×), on the number of heterozygous single nucleotide polymorphisms (SNP) which is indicative of contamination within species, and on mapped reference coverage at 5× (> 90%). Pairwise distance between isolates was calculated using SNP-sites v2.4.0 and snp-dists v0.6.3. Clusters of related isolates were identified using hierarchical clustering (Ward.D2) of 50 SNP. This more sensitive and less specific SNP threshold was chosen in the absence of a standard threshold acceptable for all *K. pneumoniae* sequence types (ST). A maximum likelihood tree was created using IQ-TREE with 1,000 bootstraps and a midpoint root based on SNP identified in 137 isolates over the entire chromosome. The maximum likelihood tree was visualised using iTOL [4]. Multilocus sequence types were identified as previously described [3]. All strains were screened for the presence of antimicrobial resistance (AMR) genes using ARIBA with the Resfinder database [5] except for 12 isolates with long-read data that were analysed using Kleborate [6]. Sequence data for all isolates have been submitted to the European Nucleotide Archive under study accession number PRJEB35890 [7].

## Phylogenetic structure

**Figure 1:**
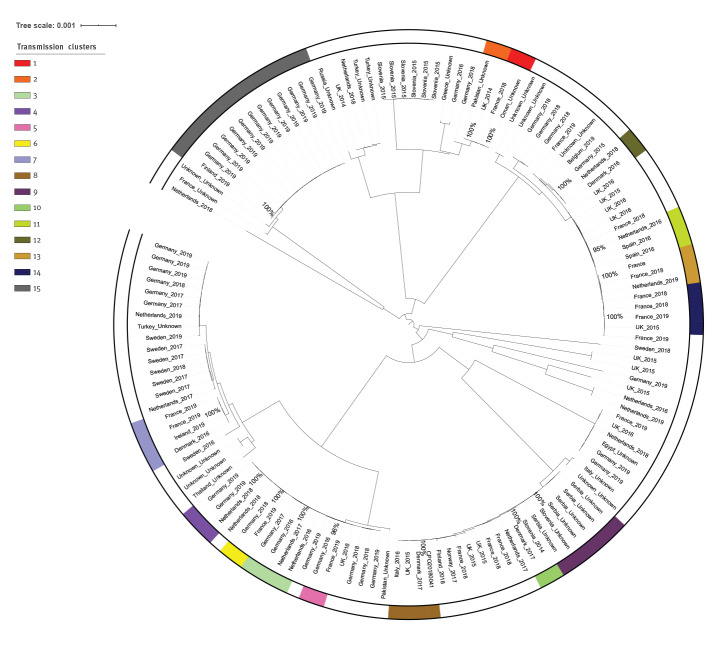
Maximum likelihood tree of *bla*_NDM-1_*-* and *bla*_OXA-48_*-*positive *Klebsiella pneumoniae* isolates based on single-nucleotide polymorphisms in whole genomes from EU/EEA national collections, 2014–2019 (n = 112), and genomes publicly available on 23 November 2019 (n = 25)

EEA: European Economic Area; EU: European Union; UK: United Kingdom.

**Figure 2:**
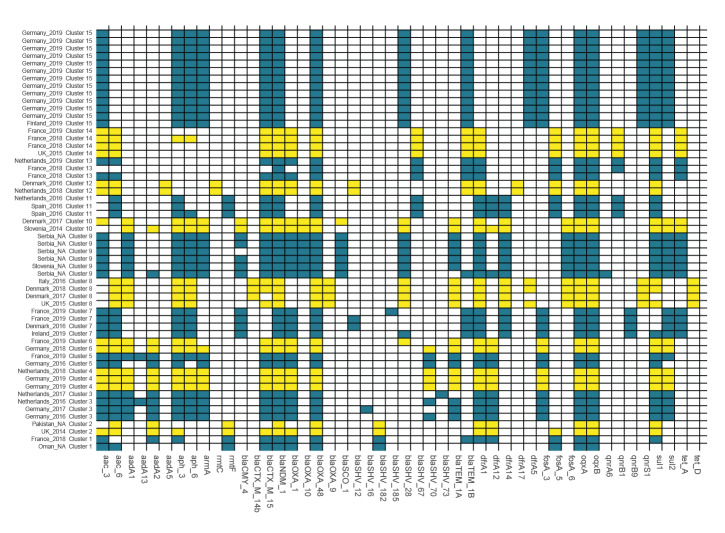
Resistome of *Klebsiella pneumoniae* isolates in clusters fulfilling the cluster definition (n = 56 isolates)

EEA: European Economic Area; EU: European Union; UK: United Kingdom.

Of the 117 isolates from national collections, three were excluded as they did not fulfil the case definition and two additional isolates were excluded because they contained a large number of heterozygous SNP sites (n = 2,498–9,766) indicating possible contamination. Of the 26 publicly accessible genomes, 18 had metadata on location of origin. After analysing the quality control of the sequences, one genome was removed because of the large number of heterozygous SNP sites (n = 650). The overall phylogenetic structure of the *K. pneumoniae* collection is presented in Figure 1. The resistome profiles were not entirely homogeneous within clusters, except for clusters 4, 12 and 15 (Figure 2).

Outer circle: clusters by colour code with white representing clusters/singletons that did not fulfil the cluster definition. The percentage bootstrap values are shown for each transmission cluster.

Including 48 genomes from national collections of EU/EEA countries, 2014–19, and 8 genomes accessed from the public domain available on 23 November 2019. Resistance genes that were positive in at least one isolate are shown on the x-axis, samples with place and year of isolation are presented on the y-axis. Clusters are highlighted in alternating colours. When the full exact amino acid match was not identified, the closest match was reported. Resistance genes for macrolides, chloramphenicol or rifampicin are not shown. *MgrB* genes were removed as truncation could only be assessed for long-read sequences which were available for very few isolates only.

## Cross-border transmission

**Figure 3:**
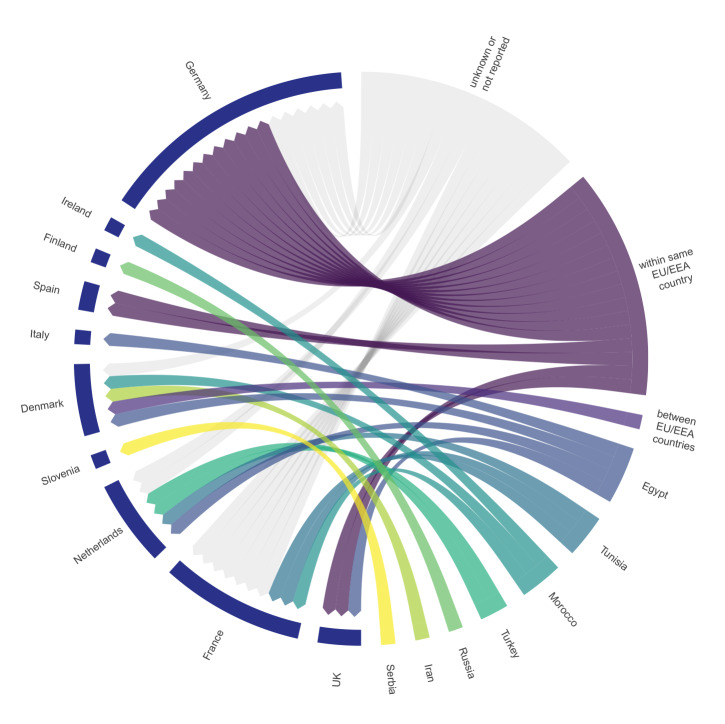
Circular diagram of epidemiological information of *Klebsiella pneumoniae *cluster isolates provided by EU/EEA countries, 2014–2019 (n = 48)

EEA: European Economic Area; EU: European Union; UK: United Kingdom.

Cross-border transmission was reported if case isolates from two or more countries were found to belong to the same specific genetic cluster, and only clusters with samples from at least two different countries are presented here. Fifteen clusters were identified that affected at least two countries, indicative of cross-border transmission. Ten of these clusters included recent isolates detected in 2018 or 2019. The size of the clusters ranged from two to 13 samples, and involved up to three different countries (Table). Of the 13 EU/EEA countries submitting national data for this analysis, 10 countries had at least one isolate in one of the cross-border clusters. For 10 clusters, epidemiological data from the patient history suggested a possible link to a country outside the EU/EEA such as Egypt, Iran, Morocco, Russia, Serbia, Tunisia and Turkey (Table and Figure 3). However, Figure 3 also indicates transmission of isolates between EU/EEA countries (1 case) and within the same country (16 cases).

**Table:** Epidemiological links and genomic characteristics of *bla*_NDM-1_- *and bla*_OXA-48_-positive *Klebsiella pneumoniae* isolates by cluster involving at least two countries (n = 15 clusters, n = 56 isolates)

Cluster number (size)	Sequence type	Countries where isolates were detected (year)	SNP distance (range)	β-lactamase resistance genes shared by all isolates	β-lactamase resistance genes not shared by all isolates	Reported history of travel or hospitalisation outside the EU/EEA
1 (n = 2)	ST11	France (2018), Oman^a^	38	*bla*_OXA-1_, *bla*_NDM-1_, *bla*_OXA-48_, *bla*_CTX-M-15_, *bla*_SHV-182_	*bla*_TEM-1B_	Tunisia (n = 1)
2 (n = 2)		UK (2014), Pakistan^a^	35	*bla*_SHV-182_, *bla*_NDM-1_, *bla*_OXA-48_	*bla*_OXA-1_, *bla*_CTX-M-15_	None
3 (n = 4)	ST14	Germany (2016, 2017), the Netherlands (2016, 2017)	0–29	*bla*_OXA-1_, *bla*_NDM-1_, *bla*_CTX-M-15_, *bla*_OXA-48_	*bla*_SHV-16_, *bla*_SHV-70_, *bla*_SHV-73_, *bla*_TEM-1A_,	Turkey (n = 1)
4 (n = 3)		Germany (2019, n = 2), the Netherlands (2018)	0–5	*bla*_OXA-1_, *bla*_NDM-1_, *bla*_OXA-48_, *bla*_CTX-M-15_, *bla*_SHV-70_, *bla*_TEM-1A_	None	Turkey (n = 1)
5 (n = 2)		France (2019), Germany (2016)	47	*bla*_NDM-1_, *bla*_OXA-48_, *bla*_CTX-M-15_, *bla*_OXA-1_	*bla*_SHV-70_, *bla*_TEM-1A_	None
6 (n = 2)		France (2019), Germany (2018)	34	*bla*_OXA-1_, *bla*_NDM-1_, *bla*_OXA-48_, *bla*_CTX-15_	*bla*_TEM-1A_, *bla*_SHV-28_, *bla*_SHV-70_	None
7 (n = 4)	ST15	Denmark (2016), France (2019; n = 2), Ireland (2019)	5–37	*bla*_OXA-1_, *bla*_CMY-4_, *bla*_NDM-1_, *bla*_OXA-48_	*bla*_SHV-12_, *bla*_SHV-28_, *bla*_SHV-185_	Morocco (n = 3)^b^
8 (n = 4)	ST101^c^	Denmark (2017, 2018), Italy (2016), UK (2015)	11–31	*bla*_SHV-28_, *bla*_OXA-9_, *bla*_NDM-1_, *bla*_OXA-48_, *bla*_TEM-1A_	*bla*_CTX-M-14b_, *bla*_CTX-M-15_	Egypt (n = 3)^b^
9 (n = 6)	ST101	Slovenia^a^, Serbia (n = 5)^a^	1–15	*bla*_SHV-28_, *bla*_OXA-10_, *bla*_SCO-1_, *bla*_NDM-1_, *bla*_OXA-48_, *bla*_CTX-M-15_	*bla*_CMY-4_, *bla*_TEM-1A_, *bla*_TEM-1B_	None
10 (n = 2)		Denmark (2017), Slovenia (2014)	19	*bla*_NDM-1_, *bla*_OXA-48_, *bla*_CTX-M-15_, *bla*_OXA-1_, *bla*_SHV-28_, *bla*_CMY-4_, *bla*_TEM-1A_, *bla*_OXA-10_	*bla*_SCO-1_	Serbia (n = 1)
11 (n = 3)	ST147	The Netherlands (2016), Spain (2016; n = 2)	15–36	*bla*_SHV-67_, bla_NDM-1_, *bla*_OXA-48_, *bla*_CTX-M-15_	None	Egypt (n = 1)
12 (n = 2)		Denmark (2016), the Netherlands (2018)	21	*bla*_OXA-1_, *bla*_NDM-1_, *bla*_OXA-48_, *bla*_CTX-M-15_, *bla*_SHV-12_, *bla*_TEM-1B_	None	Iran (n = 1)
13 (n = 3)		France (2018; n = 2), the Netherlands (2019)	21–39	*bla*_SHV-67_, *bla*_NDM-1_, *bla*_OXA-48_, *bla*_TEM-1B_	*bla*_OXA-1_, *bla*_CTX-M-15_	Tunisia (n = 2)^b^
14 (n = 4)	ST147/ST2084^c^	France (2018; n = 2), France (2019), UK (2015)	10–43	*bla*_SHV-67_, *bla*_NDM-1_, *bla*_OXA-48_, *bla*_CTX-M-15_, *bla*_OXA-1_, *bla*_TEM-1B_	None	None
15 (n = 13)	ST307	Finland (2019), Germany (2019; n = 12)	0–15	*bla*_SHV-28_, *bla*_NDM-1_, *bla*_OXA-48_, *bla_C_*_TX-M-15_, *bla*_TEM-1B_	None	Russia (n = 1)

EEA: European Economic Area; EU: European Union; SNP: single nucleotide polymorphism; UK: United Kingdom.

Including 48 genomes from national collections of EU/EEA countries, 2014–19, and 8 genomes accessed from the public domain available on 23 November 2019.

^a^ Data from the public domain; year not available.

^b^ Same country of travel or hospitalisation was reported for isolates within the cluster from at least two different EU/EEA countries.

^c^ One isolate in cluster 8 and two isolates in cluster 14 had a single nucleotide variation in one multilocus sequence typing allele (*rpoB* in cluster 8 and *mdh* in cluster 14).

Isolates from the public domain are not shown. Each line represents one case. The left part of the diagram (blue ring) shows the countries which reported cases and whole genome sequencing data. The right part of the graph shows the countries reported as places of previous hospitalisation or travel within the past six months before detection of the respective bla_NDM-1_- and bla_OXA-48_-positive *K. pneumoniae* isolate. Cases that were part of a reported national outbreak with likely patient-to-patient transmission were interpreted as transmission within the same country, unless there was available epidemiological information indicating cross-border spread.

## Discussion

The cross-border transmission or introduction of multidrug-resistant organisms (MDRO) such as *bla*_NDM-1_- and *bla*_OXA-48_*-*positive *K. pneumoniae* revealed in this study is a threat to public health and may lead to further spread within the EU/EEA as documented by recent outbreaks [1,8,9]. Our findings highlight the importance of including prior hospitalisation or recent travel to areas with a high prevalence MDRO as a risk a factor for hospital admission screening. Failing to detect carriage of MDRO may not only be detrimental for the individual patient, but also increases the likelihood of undetected transmission in the healthcare setting.

The available epidemiological information suggested several possible countries of origin for the described transmission clusters. However, the data collection was restricted to countries with the national capacity and funding to generate WGS data. Unfortunately, epidemiological evidence for a link with a non-EU/EEA country could not be substantiated by WGS results because WGS data from the potential countries of origin were not available. The evidence that epidemiologically linked countries were the sources for the respective isolates is therefore inconclusive. However, the hypothesis of potential countries of origin including Egypt, Iran, Morocco, Russia, Tunisia and Turkey is supported by the reported occurrence of *bla*_NDM-1_- and *bla*_OXA-48_*-*positive *K. pneumoniae* in those countries [10-15].

Currently established European AMR surveillance systems did not detect these multinational clusters. This study highlights the benefit of international collaboration and data sharing as these clusters were only identified by pooling of WGS data from 13 national collections. Development of WGS-based surveillance is under way with the European Antimicrobial Resistance Genes Surveillance Network (EURGen-Net), but is not yet established. The EU/EEA countries that do not perform WGS for AMR control [16,17], lack the information to elucidate cross-border transmission routes. Consequently, the identified transmission clusters may involve many more countries than described here. In addition to data sharing as part of EU-wide outbreak investigations, archiving of WGS data in the public domain provides epidemiological context for interpreting local and national data and enhances the identification of the putative sources of an outbreak with a wider benefit for AMR control.

The repeated cross-border spread of MDRO challenges the control of AMR in the EU/EEA including in countries with good detection capacity, vigorous infection prevention and control (IPC) measures and good antibiotic stewardship practices. Detection requires adequate clinical laboratory capacity to detect carbapenemase-producing Enterobacterales, sufficient WGS capacity in all EU/EEA countries to characterise the isolates and secure mechanisms for rapid sharing of WGS data at European and international level. Control requires sufficient resources for the implementation of IPC measures and cooperation with and provision of support in all these areas to countries neighbouring the EU/EEA as well as worldwide.
